# Proposed Screening for Congenital Hyperinsulinism in Newborns: Perspective from a Neonatal–Perinatal Medicine Group

**DOI:** 10.3390/jcm13102953

**Published:** 2024-05-17

**Authors:** Jeffrey R. Kaiser, Shaili Amatya, Rebecca J. Burke, Tammy E. Corr, Nada Darwish, Chintan K. Gandhi, Adrienne Gasda, Kristen M. Glass, Mitchell J. Kresch, Sarah M. Mahdally, Maria T. McGarvey, Sara J. Mola, Yuanyi L. Murray, Katie Nissly, Nanyaly M. Santiago-Aponte, Jazmine C. Valencia, Timothy W. Palmer

**Affiliations:** 1 Department of Pediatrics, Division of Neonatal-Perinatal Medicine, Penn State Health Children’s Hospital, Hershey, PA 17033, USA; samatya@pennstatehealth.psu.edu (S.A.); rburke2@pennstatehealth.psu.edu (R.J.B.); tcorr@pennstatehealth.psu.edu (T.E.C.); ndarwish@pennstatehealth.psu.edu (N.D.); cgandhi@pennstatehealth.psu.edu (C.K.G.); agasda@pennstatehealth.psu.edu (A.G.); kglass1@pennstatehealth.psu.edu (K.M.G.); mkresch@pennstatehealth.psu.edu (M.J.K.); smahdally@pennstatehealth.psu.edu (S.M.M.); mmcgarvey1@pennstatehealth.psu.edu (M.T.M.); smola@pennstatehealth.psu.edu (S.J.M.); ymurray@pennstatehealth.psu.edu (Y.L.M.); knissly@pennstatehealth.psu.edu (K.N.); nsantiagoaponte@pennstatehealth.psu.edu (N.M.S.-A.); jvalencia@pennstatehealth.psu.edu (J.C.V.); tpalmer@pennstatehealth.psu.edu (T.W.P.); 2 Department of Obstetrics and Gynecology, Penn State Milton S. Hershey Medical Center, Hershey, PA 17033, USA

**Keywords:** congenital hyperinsulinism, transitional neonatal hypoglycemia, perinatal stress-induced hyperinsulinism, screen, hypoglycemia, ketones, β-hydroxybutyrate

## Abstract

This perspective work by academic neonatal providers is written specifically for the audience of newborn care providers and neonatologists involved in neonatal hypoglycemia screening. Herein, we propose adding a screen for congenital hyperinsulinism (CHI) by measuring glucose and ketone (i.e., β-hydroxybutyrate (BOHB)) concentrations just prior to newborn hospital discharge and as close to 48 h after birth as possible, at the same time that the mandated state Newborn Dried Blood Spot Screen is obtained. In the proposed protocol, we do not recommend specific metabolite cutoffs, as our primary objective is to simply highlight the concept of screening for CHI in newborns to newborn caregivers. The premise for our proposed screen is based on the known effect of hyperinsulinism in suppressing ketogenesis, thereby limiting ketone production. We will briefly discuss genetic CHI, other forms of neonatal hypoglycemia, and their shared mechanisms; the mechanism of insulin regulation by functional pancreatic islet cell membrane K_ATP_ channels; adverse neurodevelopmental sequelae and brain injury due to missing or delaying the CHI diagnosis; the principles of a good screening test; how current neonatal hypoglycemia screening programs do not fulfill the criteria for being effective screening tests; and our proposed algorithm for screening for CHI in newborns.

## 1. Introduction

Hypoglycemia is the most common metabolic disturbance in newborns. It affects up to 15–20% of term infants and about half of at-risk newborns [1,2]. Clinicians screen for neonatal hypoglycemia in newborns with the greatest risk of impaired glucose homeostasis [3] (i.e., infants of diabetic mothers, those who are small and large for their gestational age, preterm and late preterm infants, and those with other conditions and disorders, e.g., birth asphyxia, hypothermia, family history of genetic hyperinsulinism, and maternal use of beta blockers). The purpose of screening is to identify those newborns with neonatal hypoglycemia so that they can be treated and brain injury can be averted. More than 99% of all hypoglycemic newborns have transitional neonatal hypoglycemia. Unfortunately, most screening guidelines generally identify at-risk newborns with asymptomatic transitional neonatal hypoglycemia, a condition that is not associated with long-term adverse cognitive sequelae [4]. Even though screening infants at risk for impaired metabolic transition and adaptation has been established as routine practice, it must be acknowledged that current international and local neonatal hypoglycemia screening guidelines lead to numerous painful needle sticks and sometimes the separation of infants from their parents when most of these infants do not in fact have a disease. In contrast, prolonged (perinatal stress-induced hyperinsulinism, in 1/1200 newborns) and persistent (congenital hyperinsulinism (CHI), in 1/10,000–40,000 newborns) hyperinsulinemic hypoglycemia [5] have been associated with brain injury, and up to half of infants with CHI are discharged home from their newborn hospitalization undiagnosed [6]. Most newborns with perinatal stress-induced hyperinsulinism should be identified by means of current newborn glucose screening practices because they often have symptomatic hypoglycemia and/or have risk factors. On the other hand, only about half of infants with CHI have risk factors and receive glucose screening. The objective of our proposal is to highlight the concept of screening for CHI in newborns to newborn caregivers. In this paper, we propose adding another test to the current neonatal hypoglycemia screening that focuses on identifying most infants with CHI so they can be diagnosed earlier and treated before brain damage occurs.

Unfortunately, during the first 48 h after birth, when most hypoglycemia screening occurs, it is difficult to distinguish between transitional neonatal hypoglycemia and prolonged or persistent hypoglycemia disorders [7]. For newborns who remain hypoglycemic despite frequent feeding and/or dextrose gel, intravenous dextrose infusion is the mainstay of initial treatment to prevent neuroglycopenia. When high glucose infusion rates are required to maintain euglycemia beyond 48 h after birth, prolonged or persistent hyperinsulinemic disorders need to be considered, and a comprehensive evaluation with critical labs and a glucagon challenge should be performed [7]. Detectable insulin concentrations concurrent with hypoketotic hypoglycemia as well as a robust glucose response (>30 mg/dL increase) to glucagon (1 mg) administration are diagnostic of hyperinsulinism [8,9,10].

## 2. Mechanism of Insulin Secretion

Virtually all forms of hypoglycemia in newborns are due to hyperinsulinism [5]. Transitional neonatal hypoglycemia and perinatal stress-induced hyperinsulinism likely share a common mechanism, leading to elevated insulin concentrations, albeit transiently in transitional neonatal hypoglycemia. In neonatal rat islet cells, there is a reduced pancreatic β-cell glucose threshold for insulin secretion at baseline. This is because of decreased β-cell membrane K_ATP_ channel activity, which is due to decreased trafficking of K_ATP_ channels from the cytosol to the β-cell membrane [11]. Fetal hypoxia likely plays a role in the reduced trafficking of K_ATP_ channels in transitional hypoglycemia and perinatal stress-induced hyperinsulinism. A longer duration of dysregulation of insulin secretion in infants with perinatal stress-induced hyperinsulinism may be due to a more profound and longer duration of fetal hypoxemia [12,13]. Briefly, in the normal state, glucose from a meal enters β-cells via GLUT2 glucose transporters. Glucose is then metabolized via glycolysis, the Krebs cycle, and oxidative phosphorylation, and ATP is produced. High intracellular ATP concentrations deactivate membrane K_ATP_ channels, causing them to close, thus limiting the efflux of K^+^ ions, causing membrane depolarization. Membrane depolarization opens voltage-dependent Ca^2+^ channels, increasing calcium influx and triggering insulin secretion via the exocytosis of insulin granules. Therefore, functional β-cell *membrane* K_ATP_ channels are necessary for the regulation of insulin secretion.

## 3. Transitional Neonatal Hypoglycemia and Perinatal Stress-Induced Hyperinsulinism

Transitional neonatal hypoglycemia has characteristic features of hyperinsulinism, including the suppression of ketone production, the inhibition of glycogenolysis, and an exaggerated response to glucagon; it usually resolves within 36–48 h after birth [7]. Transitional neonatal hypoglycemia is not associated with adverse neurodevelopment [4], and treatment has not been shown to confer neurocognitive benefits [14,15]. Alternatively, the relatively underrecognized perinatal stress-induced hyperinsulinism is a prolonged form of non-genetic hyperinsulinemic hypoglycemia associated with perinatal stress (e.g., perinatal depression, small for gestational age, prematurity, and preeclampsia), characterized by the requirement for a high glucose infusion rate, high responsivity to diazoxide (a drug that suppresses insulin secretion via the activation of membrane K_ATP_ channels), and eventual resolution within weeks to months after birth, confirmed by a safety fast [16,17]. Due to delayed diagnosis and treatment, infants with perinatal stress-induced hyperinsulinism are also at increased risk of neurodevelopmental abnormalities [16,18].

## 4. Congenital Hyperinsulinism: Genetics

CHI is the most common cause of persistent hypoglycemia in infants. Thirty-nine genes have been associated with CHI [5], including several syndromes. Persistent hypoglycemia in CHI is due, in a majority of patients, to monogenic variants controlling β-cell insulin secretion, most commonly as a result of inactivating mutations of the K_ATP_ channel genes, *ABCC8* and *KCNJ11*, which encode for *SUR1* and *Kir6.2* channel subunits. About 20% of patients with CHI, however, have no known identified genetic etiology [19]. Since functional membrane β-cell K_ATP_ channels are required for the normal regulation of insulin secretion, abnormal K_ATP_ channels due to genetic variants lead to uncontrolled insulin secretion and persistent hypoglycemia. In addition to low glucose concentrations, infants with CHI have characteristically suppressed ketogenesis, lipolysis, gluconeogenesis, and glycogenolysis due to the anabolic effect of insulin, and thus may have insufficient endogenous fuels that may lead to brain damage.

## 5. Congenital Hyperinsulinism: Clinical Presentation

The clinical presentation of patients with CHI is heterogeneous. A majority of patients present in the neonatal period [20,21,22,23], and the most severe cases can manifest with profound hypoglycemia with or without signs within hours of birth. Signs can range from non-specific and subtle (jitteriness and poor feeding) to severe (apnea, reduced consciousness, seizures, coma, and/or death). Many term infants with CHI are macrosomic [6,23,24], have other risk factors for hypoglycemia, and/or have signs associated with hypoglycemia [23], and are thus screened according to neonatal hypoglycemia protocols. Many others, however, are of appropriate weight for gestational age and without traditional hypoglycemic risk factors, and therefore do not receive neonatal hypoglycemia screening.

Unpublished data from the Hyperinsulinism Global Registry, a patient (family)-reported hyperinsulinism registry maintained by Congenital Hyperinsulinism International, showed that 65% (133/206) of babies with hyperinsulinism were retrospectively identified as having one or more of the risk factors identified in the American Academy of Pediatrics (AAP) neonatal hypoglycemia screening guidelines (i.e., infants of diabetic mothers, those who are small or large for gestational age, and preterm infants) [4]. Unfortunately, only 60% (80/133) of the babies with risk factors were actually identified as being at risk for hypoglycemia before leaving the birthing facility. Therefore, only 39% (80/206) of all babies with CHI had one or more of the AAP risk factors and were thus correctly identified as being at risk for hypoglycemia in the birthing facility. When maternal use of beta blockers and family history of genetic hyperinsulinism were added to the traditional AAP risk factors, only 42% (86/206) of all babies with CHI were identified as being at risk for hypoglycemia. These data from the Hyperinsulinism Global Registry show that current clinical practices are not effective in screening for CHI.

## 6. Congenital Hyperinsulinism: Classification

CHI can be classified by etiology (monogenic variants or syndromes), histopathology (diffuse, focal, or atypical), or responsiveness to diazoxide (50–60% are responsive). If the infant is diazoxide-responsive (i.e., reversal of hypoketotic hypoglycemia), diazoxide is continued and comprehensive genetic testing is done. In infants who are diazoxide-unresponsive, expedited genetic testing is performed to differentiate diffuse from focal forms. Partial pancreatectomy is curative in infants with focal disease. In diffuse disease, treatments include other medications (e.g., octreotide), continuous feeding with gastric glucose infusions, and sometimes near-total pancreatectomy.

## 7. Congenital Hyperinsulinism: Neurological-Cognitive-Developmental Disorders

Approximately 30–50% of infants with CHI still develop neurological disorders, cognitive deficits, and developmental delay due to persistent hypoglycemia and low concentrations of ketones; this is due to delayed diagnosis and treatment, late referral to a hyperinsulinism center, and failure of hypoglycemia screening protocols to identify a majority of infants with CHI [5,6,24,25,26,27,28]. Common neurological, cognitive, and developmental disorders affecting children with CHI include epilepsy, developmental delay, learning disabilities, generalized anxiety disorder, attention-deficit/hyperactivity disorder, sensory processing disorder, autism spectrum disorder, and mental health conditions [6]. The Congenital Hyperinsulinism International Hyperinsulinism Global Registry has reported that the incidence of an adverse neurological diagnosis is 20.5% when CHI is suspected by a healthcare professional during the first 48 h after birth vs. 40% when considered after this time.

## 8. Principles of an Effective Screening Test

Screening for treatable medical conditions is standard practice in perinatal medicine [29]. The purpose of screening is to identify asymptomatic individuals who have a disease in its preclinical state, whereby early treatment will ameliorate or prevent the adverse effects of the disease [30]. The screening test used to identify the disease must be safe, accurate, inexpensive, widely available, and use a widely agreed upon threshold cutoff [30]. It must have high sensitivity (low false negative rate) so that no persons with the disease will be missed and high specificity so that no person without the disease is incorrectly identified as having the disease [31]. Additionally, the test must have strong positive predictive value (low false positive rate) [31]. Other key principles of an effective screening program are that the disease for which screening is performed constitutes a public health problem, and its pathogenesis is well understood [30].

With neonatal hypoglycemia screening, we need to balance the effects of the overidentification and overtreatment of infants without a disease vs. under-identifying infants with pathological forms of hypoglycemia who are at high risk of developing brain injury if diagnosis and treatment are delayed. In the case of neonatal hypoglycemia screening, where international and local guidelines recommend that approximately one-quarter of newborns are eligible for screening [32], most of the infants identified have transitional neonatal hypoglycemia, a condition that has not been shown to have adverse neurological effects, nor has its treatment been shown to improve outcomes. The test (a blood specimen for glucose concentration) is safe and cheap, but it is not accurate, has instrument bias, and there certainly is not a consensus for a specific glucose concentration threshold cutoff for treatment [33,34]. Despite widespread targeted neonatal hypoglycemia screening, 30–50% of children with CHI still develop adverse and sometimes devastating neurological outcomes [24,25,26,27,28]. Thus, current neonatal hypoglycemia screening protocols fail to adhere to the fundamental principles of being effective screening tests [32,35], namely to identify most newborns with pathological forms of hypoglycemia before discharge from their newborn hospitalizations and before brain injury occurs [5]. Further, an effective screening test should not have unintended consequences; however, neonatal hypoglycemia screening has been associated with decreased initial exclusive breastfeeding [36]. To date, there is minimal evidence that current neonatal hypoglycemia screening programs lead to improved long-term outcomes for most infants [35,37,38].

## 9. State Newborn Dried Blood Spot Screens

The detection of serious but treatable genetic, hormone-related, and metabolic conditions before signs develop has been the purpose of mandatory state Newborn Dried Blood Spot Screening. This began in the 1960s, when Dr. Robert Guthrie first developed a simple, rapid, and cheap assay to screen for phenylketonuria using a dried blood spot on filter paper [39]. By identifying individuals with phenylketonuria early, dietary management has led to improved neurodevelopmental outcomes. Newborn Dried Blood Spot Screening has expanded dramatically over the years to include tests for inborn errors of metabolism, genetic conditions, hemoglobinopathies, and endocrinopathies, among many other disorders, and sometimes may also identify untreatable conditions [40]. Every state in the U.S. has a specific Newborn Dried Blood Spot Screening panel, aimed at detecting between 33 and 74 diseases. The incidence of individual screened disorders ranges between about 1/1500 to 1/200,000. Thus, CHI, with an incidence of 1/10,000–40,000, may be appropriate to add to state Newborn Screening panels. But even if all 39 genetic variants and syndromes associated with CHI could be identified with state Newborn Dried Blood Spot Screening, the timeframe of result reporting, often at about 7 days of age, may be too late in many infants with CHI, who may have already sustained a hypoglycemic brain injury. Therefore, another screening method should be considered for these children.

## 10. GLOW Study: Glucose and β-Hydroxybutyrate (BOHB) Concentrations

Normative BOHB data from otherwise healthy infants during the first 5 days after birth from the Glucose in Well Babies (GLOW) Study [41] can be used to support our proposed 48 h screen for CHI. Since hyperinsulinism suppresses ketogenesis, we expect that infants with CHI will have very low ketone concentrations. The GLOW Study prospectively measured glucose and alternative fuel (lactate and BOHB) concentrations longitudinally during the first 5 days after birth in term, otherwise healthy, appropriate-size-for-gestational-age, predominantly breastfed, singleton infants born to mothers without pre-pregnancy and pregnancy complications [41,42]. In this cohort, mean plasma glucose concentrations modestly increased during the first 18 h after birth, remained stable up to 48 h (59 ± 11 mg/dL), and then reached adult levels by the fourth day (83 ± 13 mg/dL) [42]. Median plasma lactate concentrations peaked within the first 12 h after birth, and subsequently decreased [41]. Median (range) BOHB concentrations increased during the first 3 days from 0.1 (0.05, 0.5) to 0.2 (0.05, 1.3), 0.6 (0.05, 2.1), and 0.7 (0.05, 2.4) mmol/L at 0–12, 12–24, 24–48, and 48–72 h, respectively, and then declined to 0.1 (0.05, 1.6) mmol/L after 96 h [41], similar to a pattern previously reported in term infants fasted for the first 24 h [43]. Stanley and colleagues [5] used normative glucose and BOHB concentrations from the GLOW Study [41,42] to propose two phases of neonatal hypoglycemia: (1) hypoketotic hypoglycemia due to hyperinsulinism from birth to 24–36 h, and (2) milder hypoglycemia with hyperketonemia due to relative fasting from insufficient and evolving breast milk production from 36 to 72 h after birth.

## 11. Proposed Algorithm for Screening for Congenital Hyperinsulinism in Newborns

**Figure 1:**
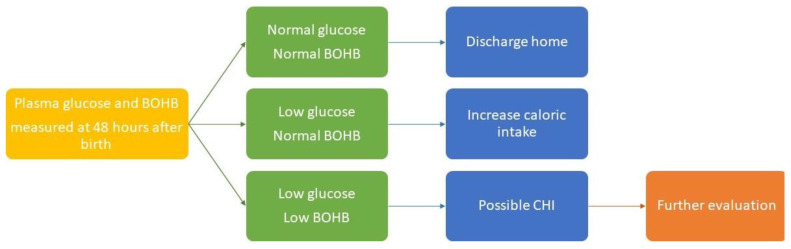
Proposed algorithm for screening for congenital hyperinsulinism in newborns. Proposed testing at the time of the state Newborn Dried Blood Spot Screen at 48 h after birth or just prior to hospital discharge. BOHB: β-hydroxybutyrate, CHI: Congenital hyperinsulinism.

Together with the state Newborn Dried Blood Spot Screen, as close as possible to 48 h after birth, we propose that clinicians screen newborns for CHI by simultaneously obtaining plasma glucose and BOHB concentrations pre-prandially, preferably using laboratory-quality bedside blood gas and chemistry analyzers, with short turnaround times. Infants with above-threshold glucose and BOHB concentrations may be discharged home. Infants with low glucose and above-threshold BOHB concentrations need increased caloric intake, and mothers should be encouraged to provide more frequent and prolonged breastfeeding using both breasts [44], or provide donor breast milk or formula, while awaiting adequate breast milk volumes. In infants with low glucose and low BOHB concentrations, CHI (as well as other forms of hyperinsulinism) should be considered and a work-up focusing on family history, physical exam, obtaining a critical sample, and a glucagon challenge test is indicated (Figure 1). While the purpose of this paper was to simply convince newborn caregivers to screen for CHI, and not to suggest specific concentration cutoffs, readers are directed to a recent article proposing a specific additive glucose plus BOHB concentration threshold (based on data from otherwise healthy breastfeeding infants from the GLOW Study [41,42]), whereby values below the threshold may indicate the presence of hyperinsulinism [45].

## 12. Limitations and Strengths of a Screen for Congenital Hyperinsulinism

There are some limitations to our proposed screening approach, as 48 h of age may actually be too late to prevent brain injury in some infants who develop profound hypoglycemia within hours of birth. Since ketone concentrations are lower in formula-fed vs. breastfed infants on the second and third days after birth [46], it is unclear whether our proposed screen will be useful in formula-fed babies, and could generate a false-positive result. Fortunately, however, a majority of infants in the U.S. receive at least some breast milk at birth [47], with even higher rates of breastfeeding in other countries, and especially in low- and middle-income countries. Another problem with our proposed 48 h screen is that at many institutions worldwide, healthy, well-feeding newborns are discharged home at ≤24 h after birth. Perhaps these infants could be screened in their primary care provider’s office at 48 h after birth during routine newborn follow-up. A final and controversial limitation is that all infants will be screened and potentially subjected to an additional painful procedure. However, according to our proposal, infants will not endure additional pain, as this screen can be performed together with the state Newborn Dried Blood Spot Screen. Given that not all newborns affected by CHI will present within the first 48 h after birth or prior to newborn hospitalization discharge, an additional limitation of our proposed screen is that a negative result could provide primary care providers with a false sense of reassurance and/or decrease their clinical suspicion for CHI as an etiology for hypoglycemia that presents after 48 h after birth. As with any rare condition, education and awareness of primary care providers as to the limitations of screening is critically important, and a negative screen should not rule out the possibility of CHI as a cause of prolonged or persistent hypoglycemia beyond 48 h of age.

The strength of our proposed screen for CHI in newborns is that a majority of infants with CHI will be identified before they are discharged home and have treatment initiated promptly. An additional benefit from our proposed screen is that infants with perinatal stress-induced hyperinsulinism and other metabolic disorders with hypoglycemia and hypoketonemia who have not been previously diagnosed will be identified before home discharge and will have treatment initiated promptly as well. Lastly, a major strength is that our proposed screen likely meets many of the principles of being a good screening test. While this test can be readily applicable, there remains a need for a large multicenter trial to determine the specific thresholds of glucose and BOHB concentrations in infants subsequently diagnosed with CHI. This would also ascertain whether our proposed screen in fact fulfills the principles for being an effective screening test, is cost effective, and ultimately reduces brain injury in these children. An additional goal of a multicenter trial will be to determine whether the threshold concentrations of BOHB in infants subsequently diagnosed with CHI are different in formula-fed vs. breastfed infants.

## 13. Conclusions

An early screen for CHI in newborns is essential to prevent hypoglycemia-induced brain injury in these infants. Including CHI as an additional disorder tested for in state Newborn Dried Blood Spot Screens will not be fully effective because many infants with CHI will have already sustained brain injury before the results become available. This paper recommends a screen to identify newborns at risk for CHI by simultaneously measuring pre-prandial glucose and BOHB concentrations at 48 h after birth, together with the state Newborn Dried Blood Spot Screen. The presence of hypoglycemia together with hypoketonemia at approximately 48 h predicts an increased risk of hyperinsulinism, in which case an evaluation for CHI should be initiated.

## Data Availability

Not applicable.

## Abbreviations

BOHB	β-hydroxybutyrate
CHI	Congenital hyperinsulinism
GLOW Study	Glucose in Well Babies Study
K_ATP_ channel	ATP-sensitive potassium channel
